# The perturbed expression of m6A in parthenogenetic mouse embryos

**DOI:** 10.1590/1678-4685-GMB-2018-0212

**Published:** 2019-11-14

**Authors:** Jindong Hao, Yu Xianfeng, Wei Gao, Jiaqi Wei, Minghui Qi, Liang Han, Shuming Shi, Chao Lin, Dongxu Wang

**Affiliations:** 1 Laboratory Animal Center, College of Animal Science, Jilin University, Changchun, China.; 2 Department of Emergency, First Hospital, Jilin University, Changchun, Jilin, China.

**Keywords:** m6A, gene expression, embryo development, parthenogenetic

## Abstract

Parthenogenetically activated oocytes cannot develop to term in mammals owing to abnormal epigenetic modifications. Methylation of the N6 position of adenosine (m6A) is a post-transcriptional epigenetic modification of RNA. To investigate the role of m6A methylation in parthenogenetic (PA) embryonic development, we analyzed *METTL3*, *METTL14*, *FTO*, *ALKBH5*, *YTHDF2, IGF2BP1,* and *IGF2BP2* expression by quantitative real-time PCR. These genes were found dynamically expressed during the 2-cell, 4-cell, 8-cell, and blastocyst stages of the embryo. Compared to normally fertilized embryos, the expression of these genes was perturbed in PA embryos, especially at the 8-cell stage. Furthermore, immunofluorescence was used to detect m6A expression. The results demonstrated that m6A expression decreased in the 2-cell stage, whereas it increased in the 8-cell stage of PA embryos. Taken together, these results suggest that the expression of RNA methylation-related genes was perturbed, leading to abnormal m6A modification during early development in PA embryos.

Parthenogenetic (PA) embryos contain exclusively maternal genomes and have been used extensively to study epigenetic profiles and determine the expression patterns of imprinted genes in human diseases (Park *et al.*, 2009; Zhang *et al.*, 2015). However, due to abnormal epigenetic modification and the absence of paternal gene expression, mammalian PA embryos cannot develop to term (Zhu *et al.*, 2003). Although previous studies have demonstrated perturbations in DNA methylation in PA embryos, studies of RNA methylation in these embryos are limited (Horii *et al.*, 2008; Park *et al.*, 2011).

A growing body of evidence indicates that global mRNA m6A levels are associated with embryonic development (Zhao *et al.*, 2017; Bertero *et al.*, 2018). Recent studies have indicated that m6A methylation is a post-transcriptional modification of RNA, which is regulated by adenosine methyltransferases and demethylases (Dominissini *et al.*, 2012; Geula *et al.*, 2015). The m6A methyltransferases include METTL3 (methyltransferase like 3) and METTL14 (Ping *et al.*, 2014; Li *et al.*, 2017). The m6A demethylases include FTO (fat mass and obesity-associated protein) and ALKBH5 (AlkB homolog 5) (Feng *et al.*, 2014; Zheng *et al.*, 2017). In addition, YTHDF2 (YTH N6-methyladenosine RNA binding protein 2) and IGF2BPs (insulin-like growth factor 2 mRNA-binding proteins) regulate mRNA fate by recognizing the consensus GG (m6A) C sequence (Nguyen *et al.*, 2014; Wang and He, 2014; Huang *et al.*, 2018).

In the present study, the expression patterns of *METTL3*, *METTL14*, *FTO*, *ALKBH5*, *YTHDF2, IGF2BP1,* and *IGF2BP2* were determined by qRT-PCR during early embryonic development. Furthermore, immunofluorescence (IF) staining was used to compare m6A expression between normally fertilized and PA embryos. The experiments involving mice were carried out in accordance with the guidelines on animal care and use of animals in research and were approved by the Animal Care and Use Committee of Jilin University, Changchun, China (No. 201706005).

To analyze embryonic development, female ICR mice (6–8 weeks old) were obtained from the School of Medical Science, Jilin University. For the superovulation test, female mice were superovulated by an intraperitoneal injection of 10IU pregnant mare’s serum gonadotropin (PMSG; Merck Millipore) followed by an intraperitoneal injection of 10IU human chorionic gonadotropin (hCG; Sigma, USA) 48 h later. Subsequently, the female mice were individually mated with ICR males with proven fertility. The females were euthanized by cervical dislocation and oviducts were removed. Two-cell stage embryos were collected into droplets of pre-equilibrated M16 media (Sigma). The 2-cell stage embryos were then washed and cultured in M16 media overlaid with mineral oil and incubated at 37 °C in a humidified atmosphere of 5% CO_2_ in the air until they developed to the 4-cell, 8-cell, and blastula stages.

The production of PA embryos was previously described (Ma *et al.*, 2005). Briefly, the oviducts of female mice were removed and cumulus-oocyte complexes (COCs) were collected, which were unfertilized oocytes after PMSG and hCG injection. The cumulus was removed and collected by briefly exposing MII oocytes to serum-free medium containing hyaluronidase (Sigma). The oocytes were treated with calcium ionophore (ionomycin Calcium salt; Sigma) for 5 min. Parthenogenesis was activated after incubation of the unfertilized oocytes in M16 medium containing 6-DMAP (2 mmol/L, Sigma) for 4h. Then, these unfertilized oocytes were transferred to fresh M16 medium. Parthenogenetic activation was confirmed by the presence of two pronuclei, which developed to the 2-cell stage. These parthenogenetic embryos (2-cell stage) were then transferred to fresh M16 medium and incubated until they reached the 4-cell, 8-cell, and blastula stages.

For qPCR analysis, total RNA was extracted from each group of embryos (n = 10) using the AllPrep DNA/RNA Micro Kit (QIAGEN, Germany) following the manufacturer’s instructions. cDNA was synthesized using the First-Strand cDNA Synthesis kit (Promega, USA). Quantitative real-time PCR (qRT-PCR) was performed to determine *METTL3*, *METTL14*, *FTO*, *ALKBH5*, *YTHDF2 IGF2BP1,* and *IGF2BP2* expression using the BioEasy SYBR Green I Real-Time PCR Kit on the BIO-RAD iQ5 Multicolor Real-Time PCR Detection System (Bioer Technology, China). The primer sequences used in this study are listed in Table S1. PCR was performed by initial denaturation at 95 °C for 3 min, followed by 40 cycles of denaturation at 95 °C for 10 s, annealing at 60 °C for 15 s, and extension at 72 °C for 30 s. The 2^-DDCT^ method was used to determine relative gene expression, which was normalized to the transcript amount of the endogenous control gene, *GAPDH*. The experiments were performed at least in triplicate. The qRT-PCR data were analyzed by *t*-tests using SPSS 16.0 (SPSS Inc., Chicago, IL, USA). A *p-*value of <0.05 was considered statistically significant.

To detected m6A expression, IF was used. Briefly, the embryos were washed three times in PBS-PVA. Then, the thinning of the zona pellucida was performed using Tyrode’s Solution (Jisskang, China). The embryos werefixedwith 4% paraformaldehyde for 30 min at room temperature (RT). After fixation, the embryos were washed with PBS-PVA and permeabilized with PBS containing 0.2% Triton X-100 for 30 min. Embryos were then incubated in PBS containing 1% bovine serum albumin(BSA) for 1 h. Next, the embryos were probed with m6A (1:500, Abcam) antibodies and incubated at 4 °C overnight. The embryos were washed with PBS three times for 10 minutes each followed by incubation with Alexa Fluor 488-conjugated secondary (anti-rabbit) antibodies for 1 h at RT. DNA was stained with 10 ng/mL Hoechst 33342 (Thermo Scientific) for 15–20 min. The embryos were washed thrice with PBS-PVA for 10 min each, air dried, and mounted on a coverslip and a glass slide using an antifade mounting medium (BOSTER, China). Images of embryos were obtained using a confocal laser scanning microscope.

To better understand m6A modification in mice parthenogenesis, we analyzed the expression patterns of m6A-associated genes by qRT-PCR at the 2-cell, 4-cell, 8-cell, and blastocyst stages of the embryos. As m6A `writers’, *METTL3* and *METTL14* methylate the N6 position of adenosine on RNAs (Sledz and Jinek, 2016). *METTL14* is believed to act synergistically with *METTL3* (Wang *et al.*, 2014). Moreover, the loss of *METTL3* expression leads to embryonic lethality in mice (Geula *et al.*, 2015). Our results showed that compared to the control (Con) embryos, *METTL3* levels were lower in the 2-cell stage (*p*<0.05) and increased in the 8-cell stage (*p*<0.05) of PA embryos. However, *METTL14* expression was low in the 4-cell (*p*<0.05) and 8-cell stage (*p*<0.05) of PA embryos (Figure 1A, B). *FTO* and *ALKBH5* are demethylases that act as `erasers’ of the m6A modification (Huang *et al.*, 2015). Previous studies of *FTO* and *ALKBH5* have indicated the reversible nature of the RNA methylation process (Jia *et al.*, 2011; Zheng *et al.*, 2013). Unlike *METTL3*, the expression of both *FTO* and *ALKBH5* increased in the 2-cell (*p*<0.05) and 8-cell stages (*p*<0.05) in the PA embryo (Figure 1C, D). *YTHDF2*, *IGF2BP1,* and *IGF2BP2* are m6A `readers’ that recognize and bind to the m6A sites on target mRNAs (Ivanova *et al.*, 2017; Huang *et al.*, 2018). It has been demonstrated that m6A selectively binds to *YTHDF2* and thus might contribute to the regulation of mRNA degradation (Chandola *et al.*, 2015). *IGF2BP*1 and *IGF2BP2* are critical for mRNA stability and translation through m^6^A modification (Huang *et al.*, 2018). In this study, the expression of *YTHDF2*, *IGF2BP1,* and *IGF2BP2* decreased in the 2-cell stage (*p*<0.01) and increased in the 8-cell stage (*p*<0.05) of PA embryos (Figure 2A–C). Thus, we demonstrate that *METTL3*, *METTL14*, *FTO*, *ALKBH5*, *YTHDF2, IGF2BP1,* and *IGF2BP2,* which are m6A methylation-related genes, are dynamically expressed during early embryonic development in mice (Kang *et al.*, 2003). Furthermore, the abnormal expression of these genes might result in inappropriate m6A methylation in PA embryos.

**Figure 1:**
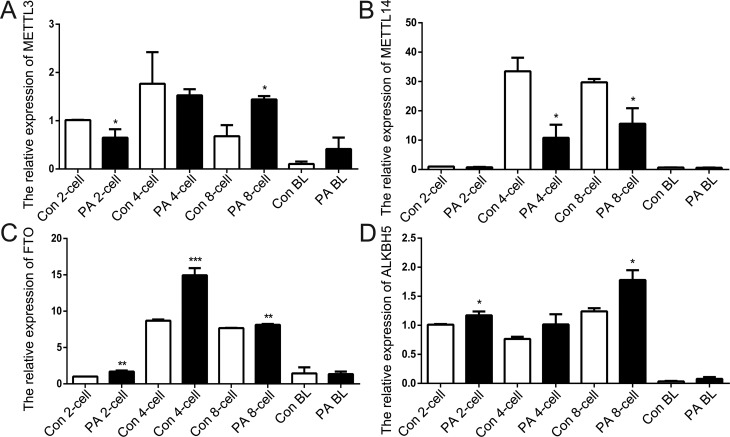
Relative expression levels of RNA methylation-related genes in both normal and PA embryos. The expression levels of *METTL3* (A), *METTL14* (B), *FTO* (C), and *ALKBH5* (D) were analyzed by qRT-PCR. The data are represented as means ± S.E.M. (n = 3). * *p*<0.05, ** *p*<0.01, *** *p*<0.005 indicate the respective statistically significant differences.

**Figure 2:**
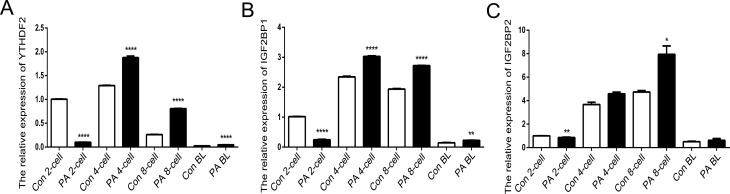
Relative expression levels of RNA methylation-related genes in normal and PA embryos. The expression levels of *YTHDF2* (A), *IGF2BP1* (B), and *IGF2BP1* (C) were analyzed by qRT-PCR. The data are represented as the mean ± S.E.M. (n = 3). * *p*<0.05), ** *p*<0.01, **** *p*<0.001 indicate the respective statistically significant differences.

RNA m6A methylation affects RNA stability, which is crucial for embryonic development (Zhao *et al.*, 2017). To further assess the effect of altered m6A methylation on PA embryonic development, we assessed m6A expression using IF staining. The results showed that compared to the Con group, m6A expression decreased in the 2-cell stage, whereas it increased in the 8-cell stage of the PA embryos (Figure 3), which is indicative of the abnormal expression of m6A in these embryos. Based on our results, we speculated that the increase in m6A expression in the 2-cell stage of control embryos was due to enhanced expression of the writer (*METTL3*) and reader (*YTHDF2*, *IGF2BP1,* and *IGF2BP2*), whereas m6A expression increased only in the 8-cell stage of the PA embryos (Figure 4). Indeed, the expression of both *METTL3* and *YTHDF2* were important for PA embryonic development. A previous study has suggested that *YTHDF2* facilitates maternal mRNA clearance in the maternal-to-zygotic transition (MZT) (Zhao *et al.*, 2017). Due to the lack of paternal genomes in PA embryos, MZT might be delayed compare to that in control embryos, which occurs at the 2-cell stage (Bouniol *et al.*, 1995). Thus, the expression of *METTL3* and *YTHDF2* might be regulated by the m6A expression pattern in the 8-cell stage of PA embryos. In addition, m6A expression was higher in the blastocyst stage than in the 2-cell, 4-cell, and 8-cell stages, which might be due to the translation of accumulated mRNAs of *METTL3* (Li *et al.*, 2017). Indeed, numerous imprinting genes are expressed and genomic imprinting is established at the mouse 2-cell stage (Kamimura *et al.*, 2014). Our results indicated that RNA methylation occurs in this stage. However, imprinted genes were abnormally expressed, and were delayed in PA embryos. This might affect RNA methylation established during PA embryonic development.

**Figure 3:**
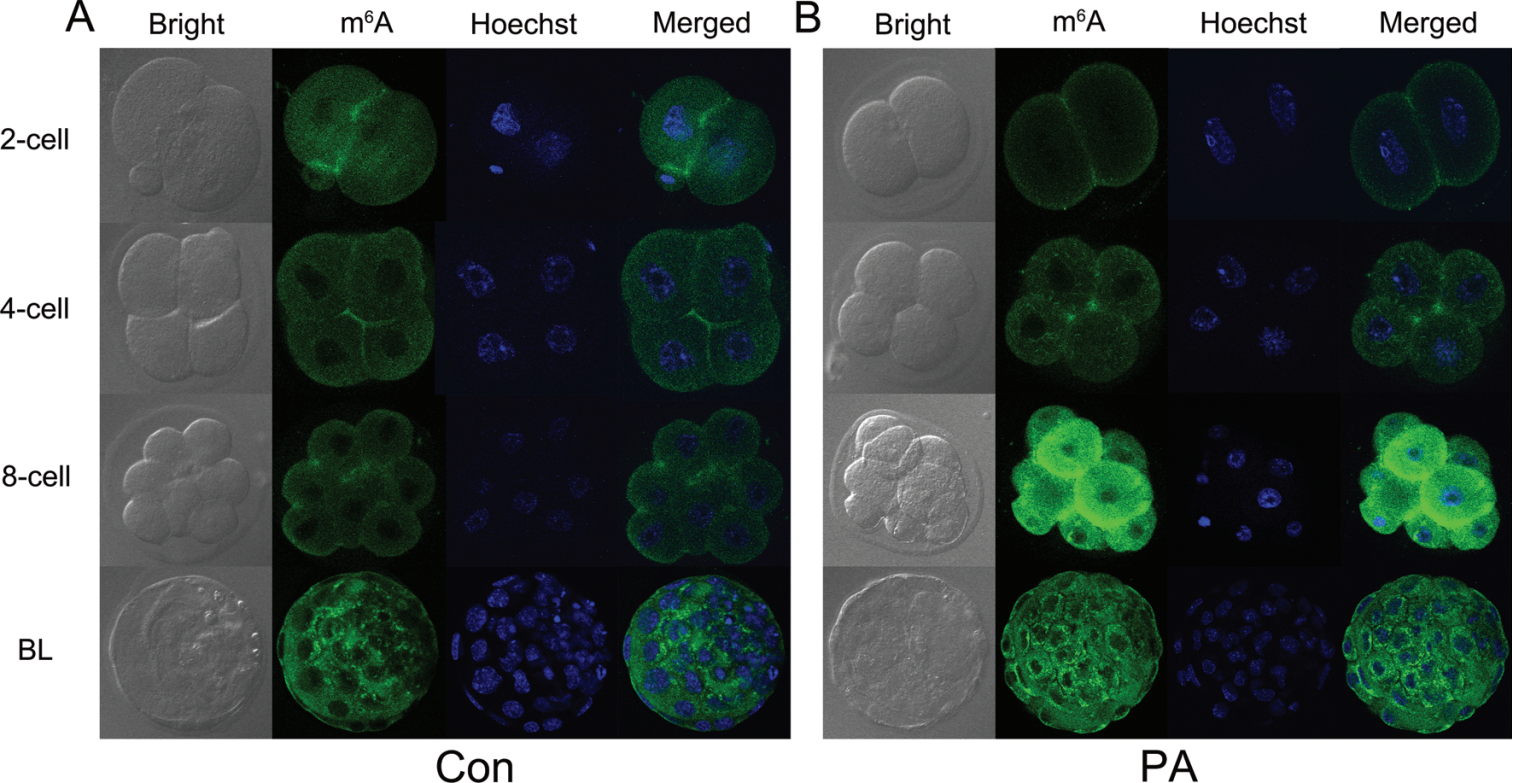
Immunofluorescence localization of m6A. The expression patterns of m6A in 2-cell, 4-cell, 8-cell, and blastocyst stages of normal (A) and PA (B) embryos. BL indicates the blastocyst stage of the embryo. Green indicates m6A. Blue indicates DNA stain.

**Figure 4:**
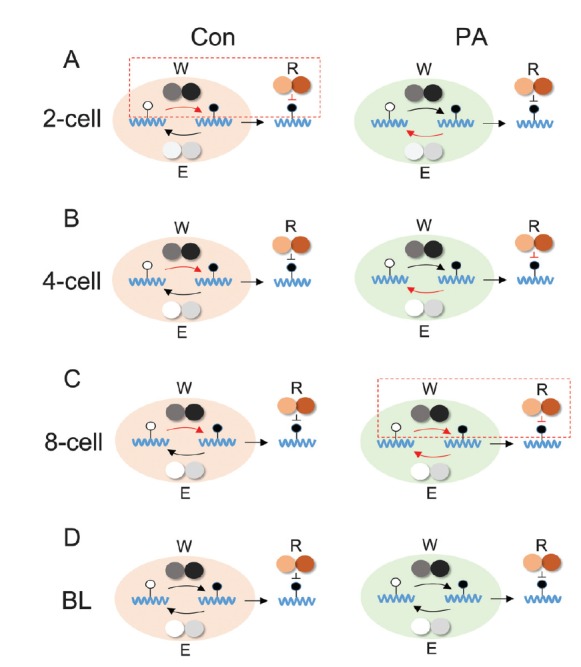
Schematic representations of m6A expression patterns in 2-cell (A), 4-cell (B), 8-cell (C), and blastocyst stage (D) of normal and PA embryos. BL indicates the blastocyst stage of the embryo. W indicates writer (*METTL3* and *METTL14*). E indicates eraser (*FTO* and *ALKBH5*). R indicates reader (*YTHDF2*, *IGF2BP1*, and *IGF2BP2*). Red arrow and inverted T indicate the over expression of genes. The red dotted line indicates enhance m6A expression.

The expression of imprinted genes and establishment of DNA methylation are dynamically regulated during early embryonic development (SanMiguel and Bartolomei, 2018). Our results confirmed that m6A methylation is perturbed in the PA genome (Sepulveda-Rincon *et al.*, 2016). As the m6A methylation level is related to gene expression, it may be responsible for developmental failure during parthenogenesis.

In conclusion, our results suggest that m6A methylation-related gene expression changes dynamically and is perturbed during the development of PA embryos. Furthermore, m6A expression was lower in the 2-cell stage, whereas it increased in the 8-cell stage of PA embryos. Therefore, the findings of the present study suggested that the development of PA embryos is characterized by abnormal m6A expression, which is associated with altered levels of m6A methylation-related genes.

## Supplementary Material

The following online material is available for this article:

Table S1 Primers for qRT-PCR analysis.
