# Supplementary material for: The perturbed expression of m6A in parthenogenetic mouse embryos
Source: Genet Mol Biol. 2019 Nov 14;42(3):666–70. doi: 10.1590/1678-4685-GMB-2018-0212 (PMC6905444; doi:10.1590/1678-4685-GMB-2018-0212)
Supplement: Supplementary file 1 [file 1415-4757-GMB-42-3-2018-0212-20190903-suppl1.pdf]

Supplementary material to: "The perturbed expression of m6A in parthenogenetic mouse embryos"

Table S1 - Primers for qRT-PCR analysis

Genes	Annealing (°C)	Primer sequences (5'→3')	Reference/accession
METTL3	60	F: GAAACAGCTGGACTCGCTTC<br>R: GGCACGGGACTATCACTACG	(Li *et al*. 2017)
METTL14	60	F: GCTAAGTCAAACACTCCTCCCA<br>R: TATTCTTCCAGAGGGGGCTC	(Li *et al*. 2017)
ALKBH5	60	F: CGCGGTCATCAACGACTACC<br>R: ATGGGCTTGAACTGGAACTTG	(Zheng *et al*. 2017)
FTO	60	F: GACACTTGGCTTCCTTACCTG<br>R: CTCACCACGTCCCGAAACAA	(Li *et al*. 2017)
YTHDF2	60	F: GAGCAGAGACCAAAAGGTCAAG<br>R: CTGTGGGCTCAAGTAAGGTTC	(Zheng *et al*. 2017)
IGF2BP1	60	F: ATCGGAGCTGAGGTGGAATA<br>R: CTCGGGGAAAGTAGAACTGC	(Nguyen *et al*., 2014)
IGF2BP2	60	F: GCAGGATCCCAGCAAATAAA<br>R: GGGCCAACAACCTCGTATAA	(Nguyen *et al*., 2014)
GAPDH	60	F: AGGTCGGTGTGAACGGATTTG<br>R: TGTAGACCATGTAGTTGAGGTCA	(Zheng *et al*. 2017)

References

Nguyen, L.H., Robinton, D.A., Seligson, M.T., Wu, L.W., Li, L., Rakheja, D., Comerford, S.A., Ramezani, S., Sun, X.K., Parikh, M.S., *et al.* (2014). Lin28b Is Sufficient to Drive Liver Cancer and Necessary for Its Maintenance in Murine Models. Cancer cell *26*, 248-261.

All other references listed in this table are in the References list of the main text.
